# Prediction models for the occurrence and mortality of sepsis-associated lung injury: a systematic review and meta-analysis

**DOI:** 10.3389/fmed.2026.1807294

**Published:** 2026-06-09

**Authors:** Chen Liu, Jian Huo, Yan-Song Li, An-Min Hu, Ting-Ting Ao

**Affiliations:** 1 Department of Anesthesiology, Renmin Hospital, Hubei University of Medicine, Shiyan, Hubei, China; 2 Shenzhen Unite Scheme Technology Co., Ltd., Shenzhen, China; 3 Department of Anesthesiology and Surgery, The First Affiliated Hospital of Xi’an Jiaotong University, Xi’an, Shaanxi, China

**Keywords:** lung injury, meta-analysis, prediction models, sepsis, systematic review

## Abstract

**Background:**

Sepsis is a life-threatening syndrome driven by dysregulated inflammation and immunity, often leading to multiple organ dysfunction. The lung is commonly affected early, and sepsis-related lung injury, including acute respiratory distress syndrome (ARDS), is associated with poor survival. Although models have been proposed to predict lung injury and short-term mortality once injury occurs, their performance, methods, and certainty of evidence remain insufficiently assessed.

**Methods:**

We searched PubMed, Embase, and the Cochrane Library for studies published up to December 11, 2025. Risk of bias and applicability were assessed with PROBAST, and certainty of evidence was appraised using an AUC-based adaptation of GRADE. The protocol was registered in PROSPERO. The individual prediction model was the unit of analysis. We extracted AUC, sensitivity, and specificity. Pooled estimates were calculated separately for ARDS occurrence and short-term mortality in sepsis-associated lung injury, and separately for training, validation, and test phases, with no pooling across phases.

**Results:**

Nine studies were included, eight of them from China. Together they reported 68 model phase units: 24 training, 21 validation, and 23 test AUCs. PROBAST classified 4 studies as having high overall risk of bias and 6 as having unclear risk; only three studies had low concern for applicability. Certainty of evidence was low for all outcome families and modeling phases. For ARDS occurrence, the pooled test-phase AUC was 0.749 (95% CI, 0.648–0.849; *I*^2^ = 98.9%). For short-term mortality, pooled AUCs were 0.800 in training (95% CI, 0.761–0.838; *I*^2^ = 97.9%), 0.778 in validation (95% CI, 0.751–0.804; *I*^2^ = 63.5%), and 0.815 in testing (95% CI, 0.780–0.850; *I*^2^ = 75.7%). Leave-one-out analyses indicated that no single study substantially changed the pooled estimates. Within each outcome family, machine learning models did not consistently outperform logistic regression. Heterogeneity remained high, particularly for ARDS occurrence and mortality training models.

**Conclusion:**

Current models showed moderate discrimination, but their clinical use is limited by bias, weak methods, low certainty, and heterogeneity. ARDS occurrence and mortality should be developed, validated, and reported separately. Future work needs transparent designs and external validation before implementation.

**Systematic review registration:**

https://www.crd.york.ac.uk/PROSPERO/view/CRD420251275870, identifier CRD42025127587.

## Introduction

Sepsis is a complex clinical syndrome triggered by infection and characterized by dysregulated systemic inflammatory responses and immune dysfunction, frequently progressing to multiple organ dysfunction and threatening patient survival (1, 2). Epidemiological estimates indicate that sepsis affects more than 19 million individuals worldwide each year (3). Given its high incidence and mortality, sepsis constitutes a major global public health challenge and remains among the leading causes of death worldwide (4). A large cross-sectional study of intensive care unit (ICU) patients reported a 3-month mortality rate of 35.5% among patients with sepsis (5). Despite advances in diagnostic strategies and therapeutic interventions, clinical outcomes for patients with sepsis remain unsatisfactory.

The lungs are among the earliest and most vulnerable organs affected during sepsis (6). Previous studies have shown that excessive inflammatory activation and endothelial dysfunction during sepsis commonly lead to pulmonary injury (7). In some patients, this process progresses to acute respiratory distress syndrome (ARDS), which substantially increases mortality risk (8, 9). ARDS is a severe and life-threatening condition characterized by profound hypoxemia and respiratory failure and is recognized as one of the most common and serious complications of sepsis (10). Consequently, sepsis complicated by ARDS represents a major contributor to mortality among ICU patients (11). However, sepsis-associated lung injury represents a broad clinical spectrum, ranging from mild interstitial edema to severe ARDS. Thus, not all patients with sepsis-induced lung dysfunction meet the strict diagnostic criteria for ARDS. This heterogeneity in disease severity and mortality outcomes complicates the evaluation of predictive models. Its pathogenesis is primarily driven by inflammation-mediated increases in vascular permeability and disruption of the alveolar-capillary barrier, resulting in impaired gas exchange (12). In addition, acute lung injury is a frequent and clinically important manifestation during the course of sepsis, adversely influencing both short-term and long-term prognosis (13).

Against this background, increasing attention has been directed toward predictive models for estimating the risk of organ injury and adverse outcomes in patients with sepsis. These models integrate multiple clinical variables through statistical or computational approaches to generate individualized risk estimates (14, 15). Existing studies have provided preliminary support for the potential utility of predictive modeling in sepsis management. For instance, Li et al. externally validated a machine learning-based prognostic model for sepsis-associated acute kidney injury and demonstrated satisfactory predictive performance (16). Likewise, Xu et al. developed a model incorporating thirteen clinical parameters to predict the occurrence of ARDS in sepsis patients, achieving good discriminative performance in internal validation (8). Nevertheless, existing studies vary considerably in design, modeling methods, predictor selection, validation strategies, and performance evaluation, limiting the comparability and reliability of available evidence. Importantly, two clinically and methodologically distinct prediction tasks have emerged: occurrence prediction, which identifies patients with sepsis at risk of developing acute lung injury or ARDS, and prognostic prediction, which estimates short-term mortality among patients with established sepsis-associated lung injury. These tasks involve different target populations, predictor sets, outcome definitions, and clinical implications; therefore, combining them into a single summary estimate may obscure meaningful differences in model performance and applicability.

In summary, sepsis complicated by lung injury is associated with high morbidity and mortality and remains a major challenge in critical care. Reliable and externally validated prediction models may enable earlier identification of high-risk patients and support timely, individualized clinical decision-making. However, no previous systematic review and meta-analysis has specifically synthesized and quantitatively evaluated prediction models for sepsis-associated lung injury. The overall predictive performance, methodological quality, and certainty of evidence of these models therefore remain unclear. Accordingly, this study aims to systematically evaluate existing prediction models for sepsis-associated lung injury, assess their methodological quality, and separately synthesize evidence for occurrence-prediction and mortality-prediction models to inform future model development and clinical application.

## Methods

This study presents a systematic review of prediction models for lung injury in patients with sepsis. The review was conducted in accordance with the Preferred Reporting Items for Systematic Reviews and Meta-Analyses (PRISMA) guidelines (17), and detailed methodological information is provided in Supplementary Table 1. The study was registered in the international registry database PROSPERO (CRD420251275870). This meta-analysis was based exclusively on previously published studies, all of which had received appropriate ethical approval. As no new data were collected and no direct human participation was involved, additional ethical approval was not required.

A comprehensive literature search was performed in PubMed, Embase, and the Cochrane Library, with the search period extending up to December 11, 2025. The search strategy incorporated key terms related to sepsis, lung injury, and prediction models. No language restrictions other than English were imposed. Detailed search strategies for each database are presented in Supplementary Table 2. Two reviewers independently screened titles and abstracts to assess study eligibility. Any disagreements were resolved through discussion with a third reviewer.

### Inclusion criteria

The inclusion criteria were as follows: (1) studies enrolling adult patients with sepsis or septic shock defined according to recognized criteria (Sepsis-2, Sepsis-3, or equivalent); (2) studies developing or validating multivariable prediction models targeting one of two prespecified outcome families: (a) occurrence of sepsis-associated lung injury, defined as the development of ALI or ARDS in patients with sepsis, diagnosed using AECC, the Berlin Definition, or the modified Berlin Definition; (b) short-term mortality among patients with sepsis-associated lung injury, defined as all-cause mortality (in-hospital, 28-, 60-, or 90-day) restricted to populations with established ARDS; (3) model performance reported using AUC, sensitivity, specificity, or calibration measures; (4) full-text English-language articles.

### Exclusion criteria

The exclusion criteria were as follows: (1) duplicate publications; (2) studies with incomplete full texts, including abstracts and study protocols; (3) studies still recruiting participants and reporting no preliminary results; (4) studies predicting generic sepsis mortality in populations not restricted to those with sepsis-associated lung injury, because these models did not address either prespecified outcome question; (5) studies in which the predicted outcome could not be clearly classified as either occurrence of sepsis-associated lung injury or mortality among patients with established ARDS.

### Data extraction

Data extraction was performed using a predesigned standardized form, and all data were compiled in Excel 2019. One author extracted the relevant information, while the second author cross-checked the data to ensure accuracy and completeness. The extracted variables from eligible studies included the first author’s name, year of publication, country, study design, sample size, patient source, predicted outcome, and details of the training, validation, and test models (Tables 1–4). The primary objective of this study was to compare the predictive performance of traditional statistical models and machine learning-based models, as well as to assess their potential clinical utility. For each model, two reviewers independently classified the predicted outcome into one of two prespecified categories: “occurrence of ARDS” or “mortality among ARDS patients,” based on the outcome definition reported in the source study. Disagreements were resolved by discussion with a third reviewer. The classification scheme was piloted on three studies before formal extraction.

**TABLE 1:** Study characteristics.

Study	Study type	Sample size	Source of patients	Outcome category
Bai et al. (25), China	Retrospective	TR: 13,474, TE: 5,775	MIMIC-IV	Occurrence (ARDS)
Chen et al. (26), China	Retrospective	6,390	MIMIC-IV	Mortality (ARDS)
Lin et al. (22), China	Retrospective	TR: 9,127, VA: 2,282	MIMIC-IV	Occurrence + Mortality
Williams et al. (27), USA	Prospective	TR: 375, VA: 250	ICU	Occurrence (ARDS)
Xu et al. (8), China	Retrospective	TR: 11,566; TE: 4,957	MIMIC-IV	Occurrence (ARDS)
Xu et al. (23), China	Retrospective	TR: 2,370; TE: 1,016	MIMIC-IV	Mortality
Yao et al. (24), China	Retrospective	57	ICU	Occurrence (ARDS)
Zhang et al. (9), China	Retrospective	TR: 905, VA: 100	MIMIC-IV	Mortality (28-d)
Zhou et al. (21), China	Retrospective	TR: 7,391, VA: 3,168	MIMIC-IV	Occurrence (ARDS)

MIMIC-IV, Medical Information Mart for Intensive Care IV; ICU, intensive care unit; TE, test; TR, train; VA, validation; ARDS, acute respiratory distress syndrome.

**TABLE 2:** Training model performance.

Study	Prediction outcome	Model	Training						
			AUC	PR	SE	SP	AC	PPV	NPV
Williams et al. (27), USA	ARDS	LR	0.88	/	0.91(0.86–0.94)	0.76(0.68–0.82)	/	0.84(0.78–0.88)	0.85(0.78–0.91)
Xu et al. (8), China	ARDS	LR	0.811(0.802–0.820)	/	0.762(0.745–0.779)	0.724(0.714–0.733)	0.732(0.724–0.740)	0.422(0.407–0.437)	0.920(0.914–0.926)
Xu et al. (23), China	Mortality	XGBoost	0.951(0.942–0.961)	/	0.848(0.82–0.876)	0.912(0.898–0.925)	/	0.784(0.754–0.814)	0.941(0.929–0.952)
		LightGBM	1.0(1.0–1.0)		1.0(1.0–1.0)	1.0(1.0–1.0)		1.0(1.0–1.0)	1.0(1.0–1.0)
		RF	1.0(1.0–1.0)		1.0(1.0–1.0)	1.0(1.0–1.0)		1.0(1.0–1.0)	1.0(1.0–1.0)
		CART	0.793(0.772–0.814)		0.75(0.716–0.783)	0.72(0.699–0.741)		0.504(0.472–0.535)	0.884(0.867–0.9)
		NB	0.831(0.811–0.852)		0.667(0.63–0.703)	0.897(0.883–0.911)		0.71(0.674–0.746)	0.877(0.861–0.892)
		LR	0.835(0.817–0.854)		0.684(0.648–0.719)	0.837(0.82–0.855)		0.614(0.578–0.649)	0.875(0.859–0.891)
Zhang et al. (9), China	Mortality (28–d)	LR	0.787(0.74–0.833)	0.592(0.516–0.672)	0.71(0.643–0.788)	0.708(0.635–0.795)	0.709(0.645–0.764)	/	/
		DT	0.705(0.664–0.768)	0.549(0.488–0.621)	0.643(0.504–0.808)	0.679(0.519–0.807)	0.666(0.615–0.717)		
		RF	0.786(0.742–0.842)	0.615(0.563–0.684)	0.681(0.57–0.776)	0.748(0.696–0.796)	0.723(0.682–0.772)		
		LightGBM	0.755(0.719–0.79)	0.594(0.533–0.649)	0.616(0.537–0.699)	0.749(0.684–0.814)	0.7(0.656–0.735)		
		AdaBoost	0.765(0.726–0.826)	0.592(0.527–0.677)	0.677(0.612–0.744)	0.722(0.649–0.805)	0.705(0.655–0.762)		
		XGBoost	0.771(0.722–0.83)	0.589(0.529–0.647)	0.676(0.573–0.773)	0.735(0.642–0.77)	0.704(0.655–0.746)		
		MLP	0.791(0.741–0.841)	0.591(0.521–0.648)	0.71(0.634–0.776)	0.708(0.633–0.779)	0.709(0.65–0.756)		
		SVM	0.792(0.76–0.84)	0.606(0.554–0.658)	0.717(0.613–0.818)	0.722(0.661–0.786)	0.72(0.675–0.767)		
Zhou et al. (21), China	ARDS	DT	0.500	/	/	/	/	/	/
		KNN	0.546						
		LR	0.753						
		NB	0.733						
		RF	0.727						
		NN	0.731						
		XGBoost	0.764						
		SVM	0.725						

ALI, acute lung injury; ARDS, acute respiratory distress syndrome; ICU, intensive care unit; LR, logistic regression; XGBoost, extreme gradient boosting; LightGBM, light gradient boosting machine; RF, random forest; CART, classification and regression tree; NB, naive bayes; DT, decision tree; AdaBoost, adaptive boosting; MLP, multi-layer perceptron; SVM, support vector machine; KNN, k-nearest neighbors; NN, neural network; AUC, area under the receiver operating characteristic curve; PR, precision; SE, sensitivity; SP, specificity; AC, accuracy; PPV, positive prediction value; NPV, negative prediction value.

**TABLE 3:** Validation model performance.

Study	Prediction outcome	Model	Validation						
			AUC	PR	SE	SP	AC	PPV	NPV
Chen et al. (26), China	Mortality (ARDS)	LR	0.771	/	/	/	/	/	/
Lin et al. (22), China	NPS-ARDS	KNN	/	/	78.3	27.3	64.6	/	/
		XGBoost			96.7	27.3	78.0		
		SVM			76.7	31.8	64.6		
		DNN			86.7	0	63.4		
		DT			91.7	4.5	68.3		
	Mortality (28-d)	KNN	/	/	46.7	75.0	67.8	/	/
		XGBoost			84.1	73.3	81.4		
		SVM			86.4	60.0	79.7		
		DNN			84.1	13.3	66.1		
		DT			79.5	40.0	69.5		
Williams et al. (27), USA	ARDS	LR	0.79	/	/	/	/	/	/
Yao et al. (24), China	ARDS	LR	0.796(0.66-0.932)	/	/	/	/	/	/
Zhang et al. (9), China	Mortality (28-d)	LR	0.785(0.742–0.828)	0.591(0.526–0.652)	0.701(0.631–0.785)	0.712(0.642–0.786)	0.707(0.655–0.746)	/	/
		DT	0.682(0.606–0.759)	0.521(0.453–0.603)	0.624(0.516–0.788)	0.659(0.551–0.774)	0.646(0.586–0.716)		
		RF	0.772(0.724–0.824)	0.598(0.520–0.671)	0.655(0.582–0.749)	0.736(0.649–0.812)	0.706(0.648–0.76)		
		LightGBM	0.750(0.706–0.812)	0.592(0.530–0.648)	0.605(0.515–0.717)	0.736(0.649–0.812)	0.698(0.652–0.746)		
		AdaBoost	0.758(0.698–0.819)	0.585(0.519–0.652)	0.680(0.569–0.749)	0.712(0.623–0.772)	0.700(0.648–0.754)		
		XGBoost	0.763(0.721–0.818)	0.575(0.508–0.642)	0.670(0.605–0.728)	0.821(0.611–0.786)	0.691(0.636–0.738)		
		MLP	0.790(0.739–0.829)	0.594(0.539–0.661)	0.706(0.615–0.788)	0.713(0.642–0.780)	0.710(0.663–0.756)		
		SVM	0.816(0.796–0.829)	0.758(0.738–0.776)	0.702(0.659–0.750)	0.825(0.804–0.839)	0.771(0.75–0.798)		

ARDS, acute respiratory distress syndrome; NPS, nonpulmonary sepsis; ALI, acute lung injury; ICU, intensive care unit; LR, logistic regression; KNN, k-nearest neighbors; XGBoost, extreme gradient boosting; SVM, support vector machine; DNN, deep neural network; DT, decision tree; RF, random forest; LightGBM, light gradient boosting machine; AdaBoost, adaptive boosting; MLP, multi-layer perceptron; AUC, area under the receiver operating characteristic curve; PR, precision; SE, sensitivity; SP, specificity; AC, accuracy; PPV, positive prediction value; NPV, negative prediction value.

**TABLE 4:** Testing model performance.

Study	Prediction outcome	Model	Testing					
			AUC	SE	SP	AC	PPV	NPV
Bai et al. (25). China	ARDS	NB	0.644(0.632-0.656)	72.67% (72.42%–72.92%)	48.75% (45.83%–51.67%)	68.89% (68.69%–69.10%)	/	/
		LR	0.653(0.641–0.667)	71.95% (70.42%–73.48%)	44.18% (43.23%–45.13%)	71.34% (71.20%–71.48%)		
		GBT	0.736(0.697–0.795)	71.43% (70.12%–72.75%)	65.29% (64.10%–66.48%)	67.19% (66.46%–68.00%)		
		AdaBoost	0.895(0.834–0.936)	78.11% (74.23%–81.23%)	78.74% (76.13%–81.35%)	70.06% (68.87%–71.28%)		
		RF	0.763(0.731–0.820)	74.92% (70.85%–78.99%)	69.38% (66.82%–72.03%)	69.16% (67.11%–71.21%)		
Chen et al. (26), China	Mortality (ARDS)	LR	0.711	/	/	/	/	/
Lin et al. (22), China	NPS-ARDS	KNN	/	53.9	45.9	53.5	/	/
		XGBoost		81.1	18.9	77.5		
		SVM		63.7	35.1	62.0		
		DNN		87.8	16.2	83.7		
		DT		92.4	10.8	87.8		
	Mortality (28-d)	KNN	/	38.4	73.9	65.2		
		XGBoost		64.5	74.2	71.8		
		SVM		40.6	78.2	69.0		
		DNN		72.5	55.8	68.4		
		DT		80.3	42.0	70.9		
Xu et al. (8), China	ARDS	LR	0.812(0.798–0.826)	0.798(0.773–0.823)	0.682(0.668–0.697)	0.705(0.692–0.718)	0.385(0.364–0.407)	0.931(0.922–0.940)
Xu et al. (23), China	Mortality	XGBoost	0.833(0.804–0.861)	0.66(0.603–0.717)	0.867(0.843–0.891)	/	0.636(0.58–0.693)	0.879(0.855–0.902)
		LightGBM	0.827(0.798–0.856)	0.717(0.663–0.771)	0.784(0.755–0.814)		0.54(0.488–0.592)	0.887(0.863–0.911)
		RF	0.846(0.818–0.874)	0.789(0.74–0.838)	0.783(0.753–0.812)		0.562(0.511–0.612)	0.913(0.891–0.935)
		CART	0.753(0.718–0.787)	0.751(0.699–0.803)	0.679(0.646–0.712)		0.452(0.406–0.499)	0.885(0.859–0.911)
		NB	0.799(0.768–0.831)	0.74(0.687–0.792)	0.746(0.715–0.777)		0.506(0.457–0.556)	0.89(0.866–0.915)
		LR	0.826(0.796–0.856)	0.774(0.723–0.824)	0.751(0.72–0.783)		0.523(0.474–0.572)	0.904(0.881–0.927)

ARDS, acute respiratory distress syndrome; NPS, nonpulmonary sepsis; NB, naive bayes; LR, logistic regression; GBT, gradient boosted tree; AdaBoost, adaptive boosting; RF, random forest; KNN, k-nearest neighbors; XGBoost, extreme gradient boosting; SVM, support vector machine; DNN, deep neural network; DT, decision tree; LightGBM, light gradient boosting machine; CART, classification and regression tree; AUC, area under the receiver operating characteristic curve; PR, precision; SE, sensitivity; SP, specificity; AC, accuracy; PPV, positive prediction value; NPV, negative prediction value.

### Risk of bias

The quality of the included studies and the certainty of evidence regarding predictive model performance were assessed using PROBAST and the GRADE extension framework for prognostic studies (18). The Prediction Model Risk of Bias Assessment Tool (PROBAST) checklist was used to evaluate risk of bias across four domains: participants, predictors, outcomes, and analysis (19). Applicability concerns were assessed across three domains: participants, predictors, and outcomes. Each item was rated as “yes,” “probably yes,” “no,” “probably no,” or “no information.” A domain was considered at high risk of bias if any item was rated as “no” or “probably no,” and the overall risk of bias was judged to be low only when all domains were assessed as low risk. Applicability ratings were reported using standard PROBAST terminology, namely “low concern,” “high concern,” or “unclear concern,” rather than the non-standard terms “low/high applicability.” Because the original GRADE framework was developed for interventional studies, certainty of evidence for predictive model performance, with AUC as the primary outcome, was assessed using the prognostic GRADE extension. Risk of bias was downgraded based on overall PROBAST results; heterogeneity, indirectness, imprecision, and publication bias were evaluated using *I*^2^ statistics, study characteristics, AUC confidence intervals, funnel plots, and Egger’s test, respectively.

### Quality of evidence

The quality of evidence was assessed using the Grading of Recommendations, Assessment, Development, and Evaluation (GRADE) framework (20). Certainty of evidence was categorized as high, moderate, low, or very low. PROBAST was used as the primary tool to assess risk of bias and applicability, whereas GRADE was used to summarize the certainty of evidence for pooled AUC-based model performance estimates. The five GRADE domains were applied as follows: risk of bias was judged based on PROBAST ratings; inconsistency was assessed according to heterogeneity and variation in AUC estimates; indirectness was evaluated by considering differences in populations, outcomes, prediction settings, and validation approaches; imprecision was judged according to the width of confidence intervals and the number of contributing studies or models; and publication bias was considered based on the number of available studies and observed reporting patterns. Final ratings were assigned after considering all five domains.

### Statistical analysis

All statistical analyses were performed using RStudio (version 2025.05.1 Build 513) with R software (version 4.5.1). Continuous variables were summarized as mean ± standard deviation, and pooled estimates were reported with corresponding 95% confidence intervals (CIs). Statistical heterogeneity was assessed using Cochran’s Q test and the I^2^ statistic. A random-effects model was applied when significant heterogeneity was detected (*P* < 0.10 and I^2^ ≥ 50%); otherwise, a fixed-effect model was used. A two-sided *P*-value < 0.05 was considered statistically significant.

The unit of analysis was the individual prediction model rather than the study, because most included studies developed multiple models using different algorithms derived from the same cohort. Pooled AUCs were calculated separately for the two prespecified outcome families, namely sepsis-associated lung injury occurrence and mortality among affected patients, and separately for the training, validation, and test phases. No pooling was conducted across outcome families or modeling phases, because these outcomes represent conceptually distinct prediction tasks and training-phase AUCs are prone to optimism and are not directly comparable with out-of-sample validation or test estimates. Training-phase results were therefore interpreted descriptively, whereas validation- and test-phase estimates were considered the main evidence of model performance. Models with training AUCs ≥ 0.99 were prespecified as potentially overfit, as near-perfect training performance may indicate limited generalizability. For models reporting both training and test results, train-to-test changes in AUC were tabulated to describe potential performance decline between development and out-of-sample assessment.

To address potential clustering of multiple models within the same study, two sensitivity analyses were conducted. First, leave-one-study-out analyses were performed to determine whether any single study substantially influenced the pooled estimates. Random-effects meta-analysis was retained as the primary approach because formal hierarchical or multilevel meta-analysis was not feasible given the limited number of included studies (*k* = 9). The certainty of evidence was assessed using an adapted GRADE framework for AUC-based prediction model evidence. Risk of bias was downgraded when most contributing models were rated as having a high or unclear risk of bias in any PROBAST domain; inconsistency was downgraded when I^2^ exceeded 75%; indirectness was downgraded when populations or predictor sets differed materially from the target clinical scenario; and imprecision was downgraded when the 95% CI of the pooled AUC crossed thresholds for both clinically useful and inadequate discrimination. Publication bias was assessed using funnel plots and Egger’s test when at least 10 models were available.

## Results

### Study retrieval

**FIGURE 1:**
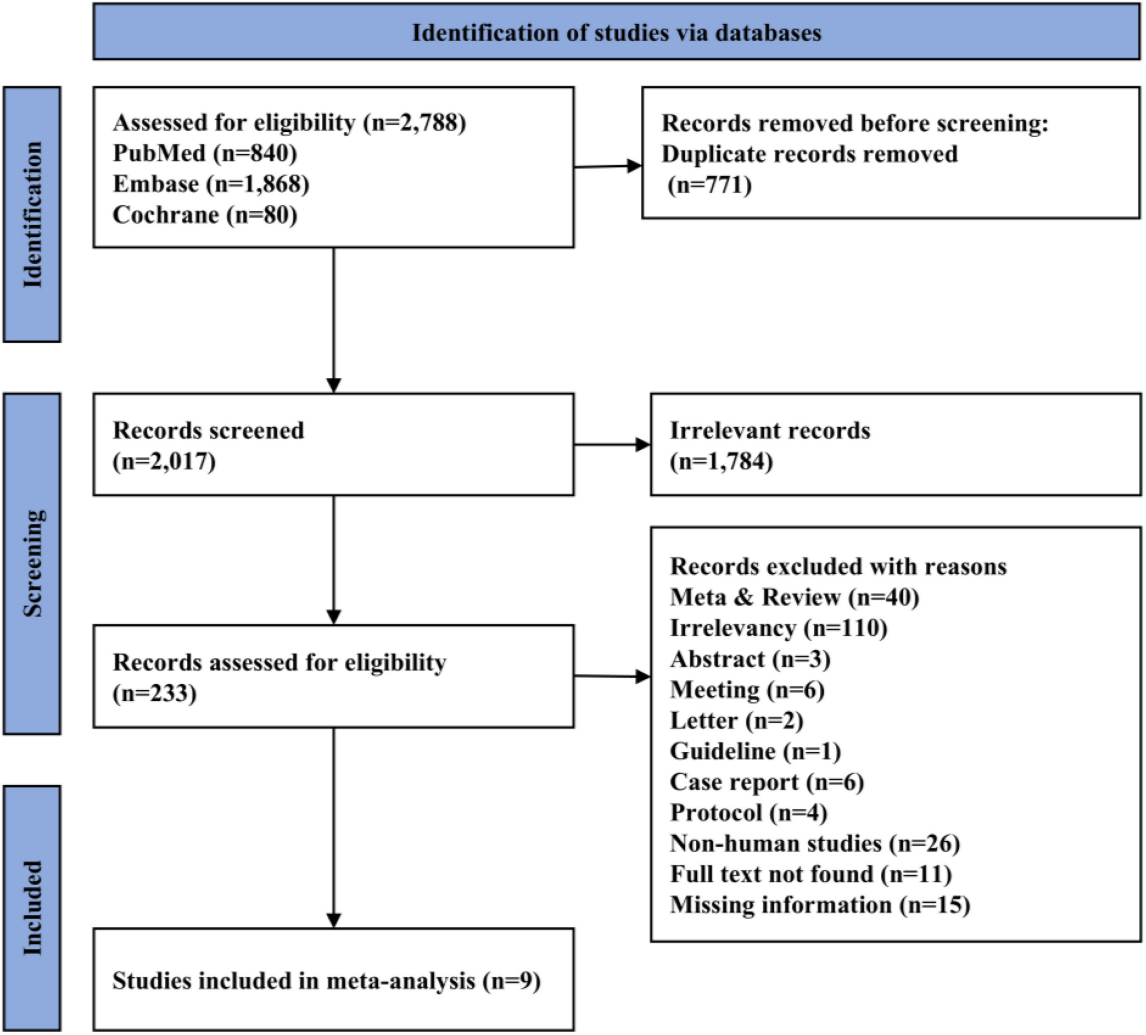
Flowchart of inclusion and exclusion criteria.

Figure 1 shows the flowchart of the study selection process. In total, we retrieved 2,788 records. After excluding 771 duplicate records, 1,784 irrelevant records, 40 meta-analyses and reviews, 110 irrelevancy, 3 abstracts, 6 meeting, 2 letters, 1 guideline, 6 case reports, 4 protocols, 26 non-human studies, 11 full texts not found, and 15 missing information. Finally, nine articles were included in the study.

### Study characteristics

Of the 9 included studies, 8 were conducted in China (8, 9, 21–26), and 1 was conducted in the United States (27). Eight studies were retrospective and 1 was prospective. The unit of analysis throughout this review was the individual prediction model. Across the 9 studies, 68 prediction model-phase units were extracted: 24 reported training-phase AUCs, 21 reported validation-phase AUCs, and 23 reported test-phase AUCs (Tables 2–4). Of these 68 model-phase units, 13 were derived from logistic regression and 55 from machine-learning algorithms. Sample sizes ranged from 57 to 19,249 participants. Each model was classified into one of two prespecified outcome families (occurrence of ARDS or mortality among ARDS patients) as detailed in Table 1. Additional baseline characteristics are summarized in Tables 1–4.

### Meta-analysis

**FIGURE 2:**
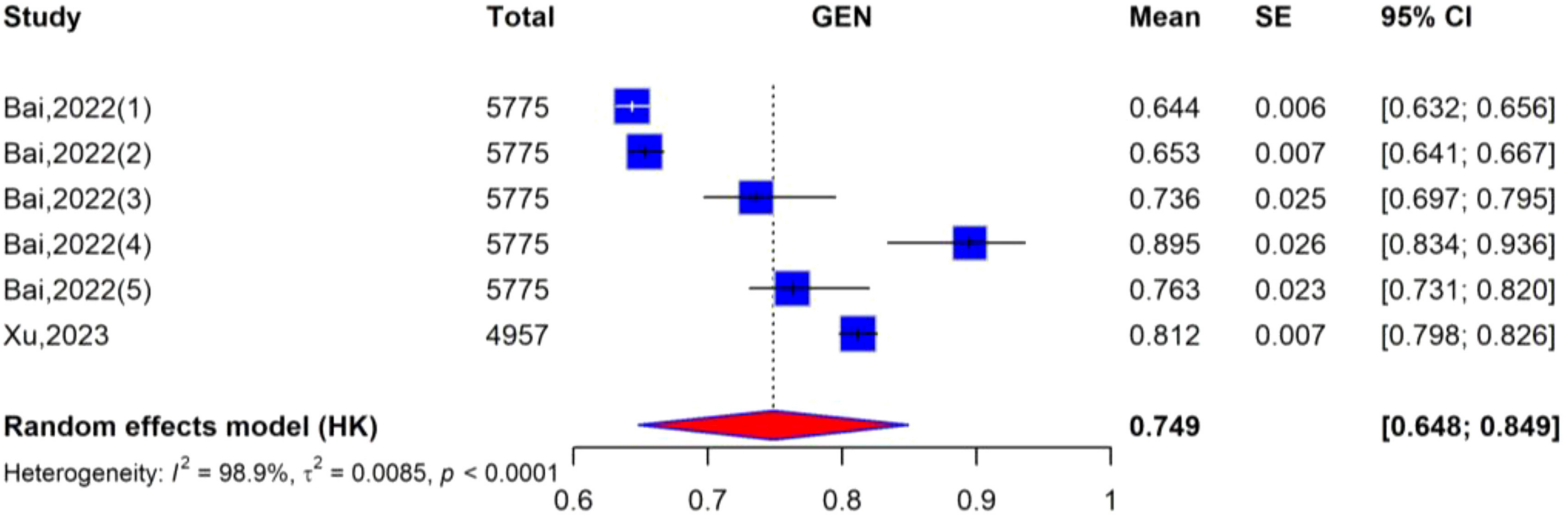
Forest plot of the random effects meta-analysis of pooled AUC estimates of test models for ARDS.

Models predicting ARDS occurrence contributed 1 training-phase, 1 validation-phase, and 6 test-phase AUCs. Pooled test AUC was 0.749 (95% CI: 0.648–0.849; *I*^2^ = 98.9%) (Figure 2). Models predicting mortality contributed 12 training-phase, 9 validation-phase, and 6 test-phase AUCs. Pooled training AUC was 0.800 (95% CI: 0.761–0.838; *I*^2^ = 97.9%), pooled validation AUC was 0.778 (95% CI: 0.751–0.804; *I*^2^ = 63.5%), pooled test AUC was 0.815 (95% CI: 0.780–0.850; *I*^2^ = 75.7%) (Supplementary Table 3). Two models within this outcome group, LightGBM and Random Forest from Xu et al. (23), reported training AUCs of 1.000, with all secondary performance metrics also equal to 1.000. Such values suggest overfitting in high-capacity ensemble tree algorithms applied without explicit regularization, and are not interpretable as estimates of generalizable predictive ability. When these two models were excluded, the mortality group training pooled AUC changed from 0.798 to 0.800. We therefore present the sensitivity-analysis estimate as the preferred summary of training-phase performance for this outcome group.

### Sensitivity analysis

**FIGURE 3:**
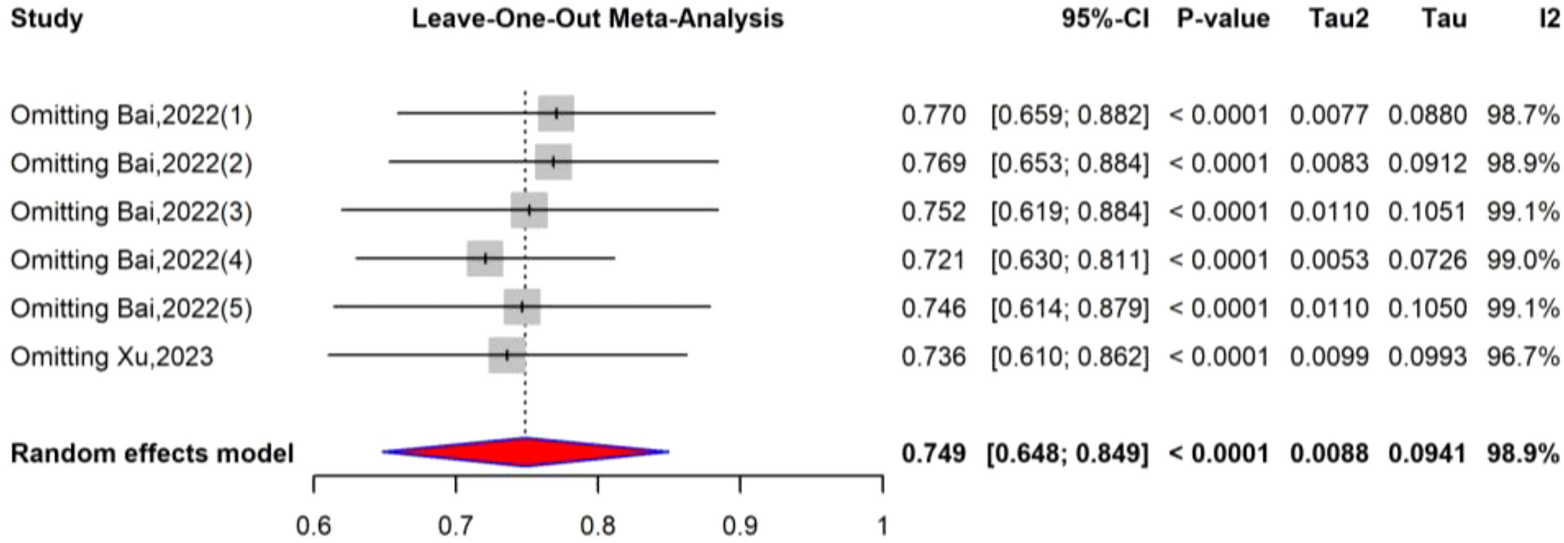
Sensitivity analysis of test models for ARDS.

Sequential leave-one-out sensitivity analyses were performed separately for ARDS occurrence and mortality prediction models. For ARDS prediction in the test phase, the overall pooled AUC was 0.749 (95% CI: 0.648–0.849; *I*^2^ = 98.9%). After omitting each study in turn, the pooled AUCs ranged from 0.721 to 0.770, and all estimates remained statistically significant (all *P* < 0.0001) (Figure 3). Although substantial heterogeneity persisted, exclusion of any single study did not materially change the pooled estimate or alter the direction of the findings. The largest shift was observed after excluding Bai, 2022(4) (25), but the change remained limited.

For mortality prediction, the pooled AUCs were generally stable across the training, validation, and test phases. In the training phase, the overall pooled AUC was 0.800 (95% CI: 0.761–0.838; *I*^2^ = 97.9%), and leave-one-out estimates ranged from 0.787 to 0.804. In the validation phase, the pooled AUC was 0.778 (95% CI: 0.751–0.804; *I*^2^ = 63.5%), with leave-one-out estimates ranging from 0.772 to 0.787. In the test phase, the pooled AUC was 0.815 (95% CI: 0.780–0.850; *I*^2^ = 75.7%), and the corresponding estimates ranged from 0.809 to 0.827. All leave-one-out estimates remained statistically significant (all *P* < 0.0001) (Supplementary Figures 1–3). Although heterogeneity persisted, no individual study materially influenced the overall pooled AUCs. The largest, but still limited, changes were observed after excluding Xu, 2025(1) in the training phase (23), Zhang, 2025(8) in the validation phase (9), and Xu, 2025(4) in the test phase (23). These findings indicate that the overall results were robust and not driven by any single study.

### Subgroup analysis

**FIGURE 4:**
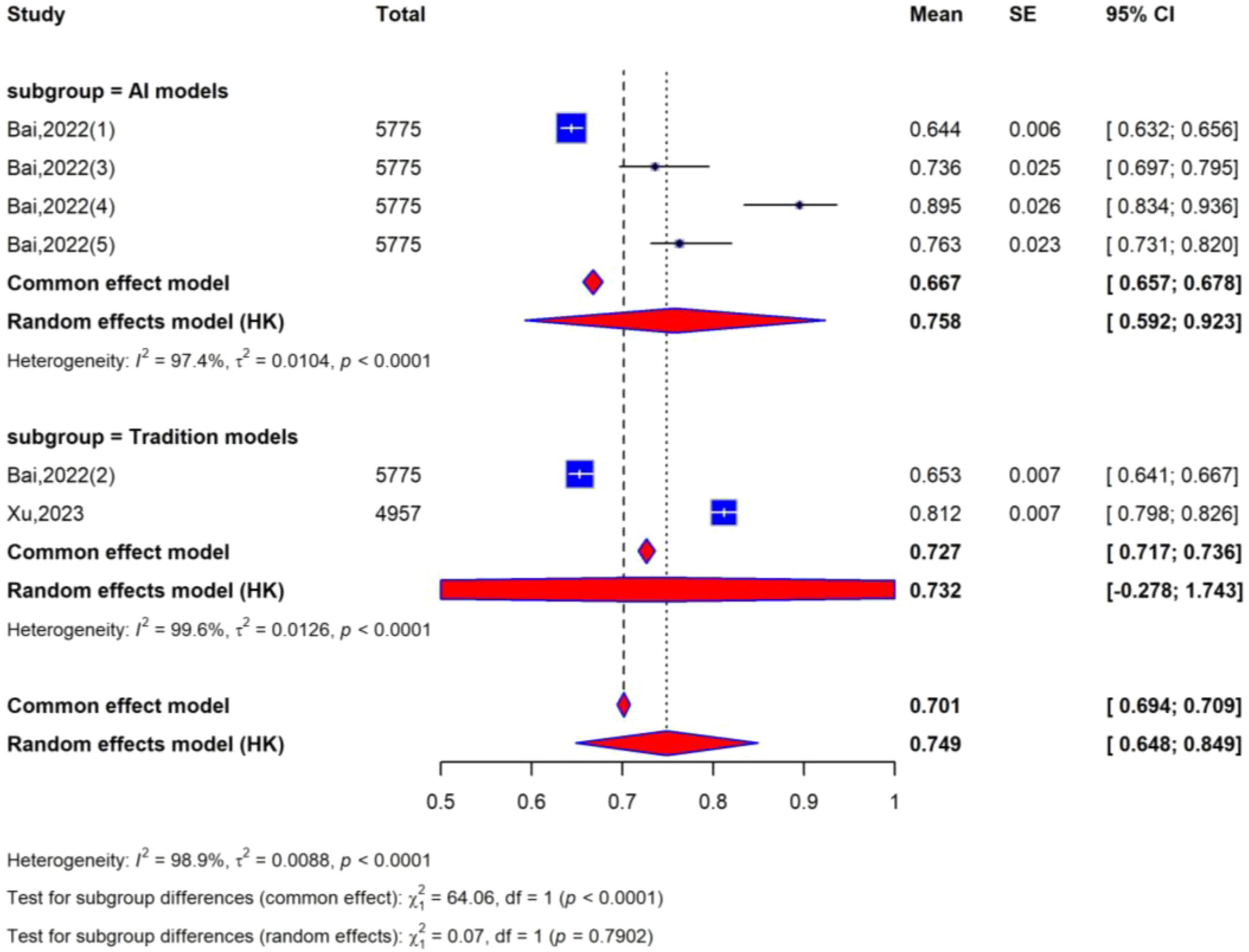
Subgroup analysis of test models for ARDS.

Subgroup analyses by model type were performed separately for ARDS occurrence and mortality prediction models. For ARDS occurrence prediction in the test phase, the pooled AUC was 0.749 (95% CI: 0.648–0.849), with substantial heterogeneity (*I*^2^ = 98.9%). Machine learning models showed a pooled random-effects AUC of 0.758 (95% CI: 0.592–0.923), whereas traditional models showed a pooled AUC of 0.732. However, the confidence interval for the traditional-model subgroup was unstable because only two models contributed to this subgroup and heterogeneity was extremely high. The random-effects test for subgroup differences showed no significant difference between machine learning and traditional models (χ^2^ = 0.07, *P* = 0.7902) (Figure 4), indicating that machine learning models did not significantly outperform traditional models in predicting ARDS occurrence.

For mortality prediction in the training phase, the overall pooled AUC was 0.800 (95% CI: 0.761–0.838), with substantial heterogeneity (*I*^2^ = 97.9%). Machine learning models yielded a pooled random-effects AUC of 0.797 (95% CI: 0.749–0.844), while traditional models showed a pooled AUC of 0.816. As in the ARDS occurrence analysis, the confidence interval for the traditional-model subgroup was unstable because only a small number of models contributed to this subgroup. The random-effects test for subgroup differences was not statistically significant (χ^2^ = 0.37, *P* = 0.5427) (Supplementary Figure 4), suggesting that machine learning models did not demonstrate a consistent performance advantage over traditional logistic regression models. Overall, subgroup analyses did not provide evidence that model type explained the observed heterogeneity or that machine learning models were superior to traditional models. Given the small number of models in several subgroups and the presence of substantial heterogeneity, these findings should be interpreted cautiously.

### Risk of bias assessment

Application of PROBAST at the model phase level identified concerns in both risk of bias and applicability (Table 5). In the Participants domain, 6 model phase units were rated as high risk and 2 as unclear. For the Predictors domain, 4 units were rated as unclear, whereas all models were assessed as low risk in the Outcome domain. In the Analysis domain, 6 units were rated as high risk and 9 as unclear. Regarding applicability, 3 models were judged as low concern and 7 as unclear in the Participants domain, while only 2 models were considered low concern in the Predictors domain. Overall, 7 models were classified as having a high risk of bias and 8 as unclear risk. In terms of overall applicability, 5 models demonstrated low concern and 5 were rated as unclear. These findings suggest that many models had unresolved methodological limitations, particularly in the analysis domain, and that future studies should use more rigorous designs, adequate validation, and more transparent reporting.

**TABLE 5:** The results of the quality assessment of included studies.

Study	ROB				Applicability			Overall	
	Participants	Predictors	Outcome	Analysis	Participants	Predictors	Outcome	ROB	Applicability
Bai et al. (25): Train model	+	+	+	?	+	+	+	?	+
Bai et al. (25): Validation model	+	+	+	?	+	+	+	?	+
Bai et al. (25): Test model	+	+	+	+	+	+	+	+	+
Chen et al. (26): Train model	−	+	+	?	−	+	+	−	−
Chen et al. (26): Validation model	?	+	+	?	?	+	+	?	?
Chen et al. (26): Test model	+	+	+	+	+	+	+	+	+
Lin et al. (22): Train model	+	+	+	?	?	+	+	?	?
Lin et al. (22): Validation model	?	+	+	?	?	+	+	?	?
Lin et al. (22): Test model	+	+	+	+	+	+	+	+	+
Williams et al. (27): Train model	+	+	+	+	+	+	+	+	+
Williams et al. (27): Validation model	+	+	+	+	+	+	+	+	+
Williams et al. (27): Test model	+	+	+	+	+	+	+	+	+
Xu et al. (8): Train model	+	?	+	?	+	+	+	?	+
Xu et al. (8): Validation model	+	?	+	+	+	+	+	+	+
Xu et al. (8): Test model	+	+	+	+	+	+	+	+	+
Xu et al. (23): Train model	+	?	+	−	?	+	+	−	?
Xu et al. (23): Validation model	+	+	+	?	?	+	+	?	?
Xu et al. (23): Test model	+	+	+	+	+	+	+	+	+
Yao et al. (24): Train model	−	?	+	−	?	−	+	−	−
Yao et al. (24): Validation model	−	+	+	−	?	−	+	−	−
Zhang et al. (9): Train model	+	+	+	?	+	+	+	?	+
Zhang et al. (9): Validation model	+	+	+	+	+	+	+	+	+
Zhang et al. (9): Test model	+	+	+	+	+	+	+	+	+
Zhou et al. (21): Train model	−	+	+	−	−	+	+	−	−
Zhou et al. (21): Validation model	−	+	+	−	−	+	+	−	−
Zhou et al. (21): Test model	−	+	+	−	+	+	+	−	+

ROB, risk of bias; Green/+, low risk of bias or low concern of applicability; Red/–, high risk of bias or high concern of applicability; Yellow/?, unclear risk of bias or unclear concern of applicability.

### Quality of evidence

The certainty of evidence was assessed using the GRADE framework. For models predicting ARDS occurrence, the training phase included 1 model from 1 study involving 11,566 patients, with a pooled AUC of 0.811 (95% CI: 0.802–0.820), sensitivity of 0.762 (95% CI: 0.745–0.779), and specificity of 0.724 (95% CI: 0.714–0.733). The validation phase included 1 model from 1 study involving 57 patients, yielding an AUC of 0.796 (95% CI: 0.660–0.932). The test phase included 6 models from 2 studies involving 10,732 patients, with an AUC of 0.749 (95% CI: 0.648–0.849), sensitivity of 0.727 (95% CI: 0.718–0.737), and specificity of 0.623 (95% CI: 0.485–0.761) (Table 6). Across the training, validation, and test phases, the certainty of evidence was rated as low because of serious concerns regarding risk of bias, inconsistency, and indirectness, although imprecision and publication bias were not considered serious.

**TABLE 6:** GRADE evidence summary for ARDS prediction.

Type		Train	Validation	Test
No. of models		1	1	6
No. of studies		1	1	2
Quality assessment	ROB	Serious	Serious	Serious
	Inconsistency	Serious	Serious	Serious
	Indirectness	Serious	Serious	Serious
	Imprecision	No	No	No
	PB	No	No	No
No. of patients		11,566	57	10,732
Results	Sensitivity (95% CI)	0.762 (0.745–0.779)	NA	0.727 (0.718–0.737)
	Specificity (95% CI)	0.724 (0.714–0.733)	NA	0.623 (0.485–0.761)
	AUC (95% CI)	0.811 (0.802–0.820)	0.796 (0.660–0.932)	0.749 (0.648–0.849)
Quality of the evidence		Low	Low	Low

ROB, risk of bias; PB, publication bias; CI, confidence interval; NA, not available; AUC, area under the receiver operating characteristic curve.

For models predicting mortality among patients with sepsis-associated lung injury, the training phase included 12 models from 2 studies involving 3,275 patients, with a pooled AUC of 0.800 (95% CI: 0.761–0.838), sensitivity of 0.729 (95% CI: 0.682–0.777), and specificity of 0.807 (95% CI: 0.752–0.861). The validation phase included 8 models from 1 study involving 100 patients, yielding an AUC of 0.778 (95% CI: 0.751–0.804), sensitivity of 0.681 (95% CI: 0.655–0.707), and specificity of 0.801 (95% CI: 0.759–0.842). The test phase included 6 models from 1 study involving 1,016 patients, with an AUC of 0.815 (95% CI: 0.780–0.850), sensitivity of 0.742 (95% CI: 0.696–0.789), and specificity of 0.777 (95% CI: 0.710–0.844) (Supplementary Table 4). Similar to the ARDS occurrence models, the certainty of evidence for mortality prediction was rated as low across all modeling phases, mainly due to serious risk of bias, inconsistency, and indirectness. Overall, the GRADE assessment indicated low certainty of evidence for both ARDS occurrence and mortality prediction models.

## Discussion

This systematic review and meta-analysis evaluated the performance of prediction models for sepsis-associated lung injury across two clinically distinct outcome families: prediction of ARDS occurrence and prediction of short-term mortality among patients with sepsis-associated lung injury. Overall, the pooled AUCs ranged from 0.749 to 0.815 across the main analyses, indicating moderate discriminatory ability and suggesting that current models have potential predictive value; however, their performance remains insufficient to support routine clinical implementation without further external validation.

Previous studies have suggested that an AUC greater than 0.80 generally indicates good and potentially clinically useful discriminatory ability, whereas an AUC of 0.70–0.80 reflects acceptable to good discrimination, and an AUC below 0.70 is usually considered insufficient for clinical decision support (28, 29). Based on these commonly used thresholds, models predicting ARDS occurrence showed acceptable but not strong discrimination, with a pooled test-phase AUC of 0.749. Although the leave-one-out sensitivity analysis indicated that no single study materially altered the pooled estimate, heterogeneity remained substantial, suggesting that the pooled result was statistically stable but should still be interpreted with caution. Differences in ARDS diagnostic criteria, patient populations, sample sizes, predictor definitions, modeling strategies, and validation approaches may have contributed to the observed variability in model performance. For models predicting mortality among patients with sepsis-associated lung injury, the pooled AUCs were 0.800 in the training phase, 0.778 in the validation phase, and 0.815 in the test phase. These values suggest moderate to good discriminatory ability overall. Although the test-phase AUC for mortality prediction was numerically higher than that for ARDS occurrence prediction, direct comparison between these two outcome categories is not statistically appropriate because they involve different clinical prediction tasks and patient populations. Therefore, models predicting ARDS occurrence and those predicting mortality should be interpreted separately rather than combined into a single prediction framework.

Subgroup analyses by model type did not demonstrate a clear advantage of machine learning over traditional statistical models. For ARDS occurrence prediction in the test phase, machine learning models showed a slightly higher pooled AUC than traditional models, but the subgroup difference was not statistically significant. For mortality prediction in the training phase, traditional models showed a slightly higher pooled AUC than machine learning models, again without a significant subgroup difference. This suggests that, despite the theoretical advantages of machine learning for modeling nonlinear relationships, superior performance cannot be assumed without adequate sample size, high-quality predictors, appropriate regularization, and robust external validation (30). Given the small number of models in several subgroups, heterogeneous study designs, and incomplete reporting, these findings should be interpreted descriptively rather than as definitive evidence of comparative model superiority (31, 32).

A recurring concern was the potential decline or instability of model performance when moving from training to validation or test settings (33). This pattern may reflect overfitting, differences in case mix, or heterogeneity in data sources across development and validation datasets (34). In the present analysis, ARDS occurrence models showed pooled sensitivities of 0.762 in the training phase and 0.727 in the test phase, while mortality models showed pooled sensitivities of 0.729, 0.681, and 0.742 in the training, validation, and test phases, respectively. These results suggest that some high-risk patients may remain undetected, especially when models are applied outside the development dataset. Although several models achieved moderate specificity, their overall generalizability remains uncertain.

Marked heterogeneity was observed across several analyses, particularly for ARDS test models and mortality training models. Leave-one-out sensitivity analyses showed that sequential exclusion of individual studies did not substantially change the pooled estimates, supporting the robustness of the main findings. However, the persistence of high heterogeneity suggests that the pooled AUCs should be regarded as approximate summaries rather than precise estimates of model performance. This pronounced heterogeneity is likely multifactorial, arising from differences in patient populations, unequal sample sizes, variability in predictor selection, heterogeneity in modeling techniques, and inconsistent definitions of study outcomes (35–37). The 95% confidence intervals were relatively wide in some analyses and subgroups, reflecting uncertainty in performance estimates (38, 39). Limited sample sizes in validation or test datasets may further reduce model stability and restrict generalizability across clinical settings (40, 41).

Overfitting was another important methodological issue. Two ensemble tree-based models in Xu et al. (23) reported perfect training AUCs of 1.000, with all secondary performance metrics also equal to 1.000 (23). Such values are unlikely to represent true generalizable predictive ability and are more consistent with overfitting in high-capacity learning algorithms, particularly when sufficient regularization or external validation is lacking. The distinction between ARDS occurrence prediction and mortality prediction is clinically important. ARDS occurrence is closely related to inflammatory and physiological derangements during sepsis (42), which may be captured by routinely available laboratory and gas-exchange variables (43, 44). In contrast, short-term mortality among patients with sepsis-associated lung injury is influenced by a broader range of factors, including baseline severity, organ dysfunction, treatment strategies, secondary complications, and healthcare resource availability (45, 46). These differences reinforce the need to develop, validate, and report models for these two prediction tasks separately. The limited reporting of lung injury onset, index time points, and prediction windows also prevented quantitative time-based subgroup analyses; therefore, potential temporal effects could only be considered qualitatively.

The PROBAST assessment revealed high or unclear risk of bias in several model-phase units, particularly in the participants and analysis domains. These concerns may reflect non-representative patient selection, retrospective study designs, inadequate handling of missing data, limited adjustment for overfitting, insufficient sample size, or incomplete reporting of model development and validation. Applicability concerns were also present, with only a subset of models demonstrating low concern for applicability. These methodological limitations reduce confidence in the reliability and transferability of existing prediction models to broader clinical settings.

The GRADE assessment further showed that the certainty of evidence was low for both ARDS occurrence and mortality prediction models across modeling phases. The main reasons for downgrading were risk of bias, inconsistency, and indirectness. Although imprecision and publication bias were not considered serious in the GRADE assessment, the limited number of studies and models within several strata still reduced confidence in the pooled estimates. Thus, despite moderate discriminatory performance, the current evidence base remains insufficient for routine clinical use.

Several limitations should be acknowledged. First, the included studies were geographically concentrated. Most were conducted in China, and only one was conducted in the United States, which may limit the generalizability of the findings to other populations and healthcare systems. Second, most studies used retrospective designs, which may increase the risk of selection bias, missing data, and inconsistent predictor measurement. Third, multiple models were often developed from the same patient cohort, and treating individual models as analytic units may introduce within-study correlation. We addressed this issue using leave-one-study-out sensitivity analyses and analyses restricted to representative models, but formal hierarchical or multilevel meta-analysis was not feasible because the number of included studies was small. Fourth, substantial heterogeneity remained despite stratification by outcome family and modeling phase. Fifth, inconsistent reporting of ARDS diagnostic criteria, mortality time windows, lung injury onset, index time points, and prediction windows limited more detailed subgroup analyses.

There were also limitations in the subgroup analyses. Several subgroups, especially those involving traditional models, included only a small number of prediction models. In these settings, Hartung-Knapp random-effects confidence intervals were unstable and sometimes extended beyond the theoretical AUC range of 0–1. This does not mean that the true AUC could fall outside this range. Rather, it reflects the imprecision caused by pooling very few models in the presence of substantial heterogeneity. Future studies with larger sample sizes, external validation, and more standardized reporting are needed to allow more reliable comparisons between traditional statistical models and machine learning approaches.

In summary, existing prediction models for sepsis-associated lung injury showed moderate discrimination for both ARDS occurrence and mortality prediction. However, their clinical use remains limited by methodological weaknesses, substantial heterogeneity, high or unclear risk of bias, and low certainty of evidence. Machine learning models did not consistently outperform traditional models, and some high-capacity algorithms showed signs of overfitting. Future studies should be prospective, multicenter, and geographically diverse, with standardized outcome definitions and prediction windows, transparent reporting of model development and validation, and rigorous external validation before clinical implementation.

## Conclusion

This review included 9 studies and 68 model-phase units covering ARDS occurrence and short-term mortality in sepsis-associated lung injury. Overall, models showed only moderate validation/test discrimination, with limited clinical applicability due to methodological flaws, high or unclear bias, applicability concerns, low certainty, and heterogeneity. Machine learning did not consistently outperform logistic regression, and two perfect training AUCs suggested overfitting. Future prospective, multicenter, externally validated studies with standardized outcomes, transparent reporting, PROBAST assessment, and TRIPOD+AI adherence are needed before bedside use.

## Data Availability

The original contributions presented in the study are included in the article/Supplementary material, further inquiries can be directed to the corresponding authors.
