# Supplementary material for: Prediction models for the occurrence and mortality of sepsis-associated lung injury: a systematic review and meta-analysis
Source: Front Med (Lausanne). 2026 Jun 9;13:1807294. doi: 10.3389/fmed.2026.1807294 (PMC13286850; doi:10.3389/fmed.2026.1807294)
Supplement: Supplementary file 1 [file Table_1.doc]

**Prediction Models for the Occurrence and Mortality of Sepsis-Associated Lung Injury: A Systematic Review and Meta-Analysis**

SUPPLEMENTAL MATERIAL

Supplementary Table 1 PRISMA Checklist

Supplementary Table 2 Search strategy

Supplementary Table 3 Forest plot of the random effects meta-analysis of pooled AUC estimates of models for mortality

Supplementary Figure 1 Sensitivity analysis of train models for mortality

Supplementary Figure 2 Sensitivity analysis of validation models for mortality

Supplementary Figure 3 Sensitivity analysis of test model for mortality

Supplementary Figure 4 Subgroup analysis of train model for mortality

Supplementary Table 4 GRADE evidence summary for mortality prediction

Supplementary Note 1 Overview of core algorithms

Supplementary Reference

Supplementary Table 1 PRISMA Checklist

| **Section and Topic** | **Item #** | **Checklist item** | **Location where item is reported** |
| --- | --- | --- | --- |
| **TITLE** | | |  |
| Title | 1 | Identify the report as a systematic review. | 1 |
| **ABSTRACT** | | |  |
| Abstract | 2 | See the PRISMA 2020 for Abstracts checklist. | 1 |
| **INTRODUCTION** | | |  |
| Rationale | 3 | Describe the rationale for the review in the context of existing knowledge. | 2 |
| Objectives | 4 | Provide an explicit statement of the objective(s) or question(s) the review addresses. | 2 |
| **METHODS** | | |  |
| Eligibility criteria | 5 | Specify the inclusion and exclusion criteria for the review and how studies were grouped for the syntheses. | 3 |
| Information sources | 6 | Specify all databases, registers, websites, organisations, reference lists and other sources searched or consulted to identify studies. Specify the date when each source was last searched or consulted. | 3 |
| Search strategy | 7 | Present the full search strategies for all databases, registers and websites, including any filters and limits used. | 3 |
| Selection process | 8 | Specify the methods used to decide whether a study met the inclusion criteria of the review, including how many reviewers screened each record and each report retrieved, whether they worked independently, and if applicable, details of automation tools used in the process. | 3 |
| Data collection process | 9 | Specify the methods used to collect data from reports, including how many reviewers collected data from each report, whether they worked independently, any processes for obtaining or confirming data from study investigators, and if applicable, details of automation tools used in the process. | 3 |
| Data items | 10a | List and define all outcomes for which data were sought. Specify whether all results that were compatible with each outcome domain in each study were sought (e.g. for all measures, time points, analyses), and if not, the methods used to decide which results to collect. | 3 |
|  | 10b | List and define all other variables for which data were sought (e.g. participant and intervention characteristics, funding sources). Describe any assumptions made about any missing or unclear information. | 3 |
| Study risk of bias assessment | 11 | Specify the methods used to assess risk of bias in the included studies, including details of the tool(s) used, how many reviewers assessed each study and whether they worked independently, and if applicable, details of automation tools used in the process. | 3 |
| Effect measures | 12 | Specify for each outcome the effect measure(s) (e.g. risk ratio, mean difference) used in the synthesis or presentation of results. | 4 |
| Synthesis methods | 13a | Describe the processes used to decide which studies were eligible for each synthesis (e.g. tabulating the study intervention characteristics and comparing against the planned groups for each synthesis (item #5)). | 4 |
|  | 13b | Describe any methods required to prepare the data for presentation or synthesis, such as handling of missing summary statistics, or data conversions. | 4 |
|  | 13c | Describe any methods used to tabulate or visually display results of individual studies and syntheses. | 4 |
|  | 13d | Describe any methods used to synthesize results and provide a rationale for the choice(s). If meta-analysis was performed, describe the model(s), method(s) to identify the presence and extent of statistical heterogeneity, and software package(s) used. | 4 |
|  | 13e | Describe any methods used to explore possible causes of heterogeneity among study results (e.g. subgroup analysis, meta-regression). | 4 |
|  | 13f | Describe any sensitivity analyses conducted to assess robustness of the synthesized results. | 4 |
| Reporting bias assessment | 14 | Describe any methods used to assess risk of bias due to missing results in a synthesis (arising from reporting biases). | 4 |
| Certainty assessment | 15 | Describe any methods used to assess certainty (or confidence) in the body of evidence for an outcome. | 3-4 |
| **RESULTS** | | |  |
| Study selection | 16a | Describe the results of the search and selection process, from the number of records identified in the search to the number of studies included in the review, ideally using a flow diagram. | 4 |
|  | 16b | Cite studies that might appear to meet the inclusion criteria, but which were excluded, and explain why they were excluded. | 4 |
| Study characteristics | 17 | Cite each included study and present its characteristics. | 4 |
| Risk of bias in studies | 18 | Present assessments of risk of bias for each included study. | 10-11 |
| Results of individual studies | 19 | For all outcomes, present, for each study: (a) summary statistics for each group (where appropriate) and (b) an effect estimate and its precision (e.g. confidence/credible interval), ideally using structured tables or plots. | 5-7 |
| Results of syntheses | 20a | For each synthesis, briefly summarise the characteristics and risk of bias among contributing studies. | 8 |
|  | 20b | Present results of all statistical syntheses conducted. If meta-analysis was done, present for each the summary estimate and its precision (e.g. confidence/credible interval) and measures of statistical heterogeneity. If comparing groups, describe the direction of the effect. | 8-10 |
|  | 20c | Present results of all investigations of possible causes of heterogeneity among study results. | 10 |
|  | 20d | Present results of all sensitivity analyses conducted to assess the robustness of the synthesized results. | 9-10 |
| Reporting biases | 21 | Present assessments of risk of bias due to missing results (arising from reporting biases) for each synthesis assessed. | 10-12 |
| Certainty of evidence | 22 | Present assessments of certainty (or confidence) in the body of evidence for each outcome assessed. | 10-12 |
| **DISCUSSION** | | |  |
| Discussion | 23a | Provide a general interpretation of the results in the context of other evidence. | 12 |
|  | 23b | Discuss any limitations of the evidence included in the review. | 12-13 |
|  | 23c | Discuss any limitations of the review processes used. | 13 |
|  | 23d | Discuss implications of the results for practice, policy, and future research. | 13 |
| **OTHER INFORMATION** | | |  |
| Registration and protocol | 24a | Provide registration information for the review, including register name and registration number, or state that the review was not registered. | 3 |
|  | 24b | Indicate where the review protocol can be accessed, or state that a protocol was not prepared. | 3 |
|  | 24c | Describe and explain any amendments to information provided at registration or in the protocol. | 3 |
| Support | 25 | Describe sources of financial or non-financial support for the review, and the role of the funders or sponsors in the review. | 14 |
| Competing interests | 26 | Declare any competing interests of review authors. | 14 |
| Availability of data, code and other materials | 27 | Report which of the following are publicly available and where they can be found: template data collection forms; data extracted from included studies; data used for all analyses; analytic code; any other materials used in the review. | 13 |

*From:*  Page MJ, McKenzie JE, Bossuyt PM, Boutron I, Hoffmann TC, Mulrow CD, et al. The PRISMA 2020 statement: an updated guideline for reporting systematic reviews. BMJ 2021;372:n71. doi: 10.1136/bmj.n71. This work is licensed under CC BY 4.0. To view a copy of this license, visit <https://creativecommons.org/licenses/by/4.0/>

Supplementary Table 2 Search strategy

| Database | Search strategy |
| --- | --- |
| PubMed | ("acute respiratory distress syndrome"[tiab] OR ARDS[tiab] OR "Acute Lung Injury"[tiab] OR "lung injury"[tiab] OR "pulmonary injury"[tiab] OR "postoperative pulmonary complications"[tiab]) AND ("Sepsis"[Mesh] OR sepsis[tiab] OR septicemia[tiab] OR "bloodstream infection"[tiab] OR bacteremia[tiab]) AND (predict*[tiab] OR "risk model"[tiab] OR "predictive model"[tiab] OR "prognostic model"[tiab] OR "early prediction"[tiab] OR "early detection"[tiab]) |
| Embase | ('acute respiratory distress syndrome':ti,ab OR ards:ti,ab OR 'acute lung injury':ti,ab OR 'lung injury':ti,ab OR 'pulmonary injury':ti,ab) AND ('sepsis'/exp OR sepsis:ti,ab OR septicemia:ti,ab OR 'bloodstream infection':ti,ab OR bacteremia:ti,ab) AND (predict*:ti,ab OR 'predictive model':ti,ab OR 'early prediction':ti,ab) |
| Cochrane | ("acute respiratory distress syndrome" OR ARDS OR "acute lung injury" OR "lung injury" OR "pulmonary injury" OR "postoperative pulmonary complications") AND ("sepsis" OR "septicemia" OR "bloodstream infection" OR "bacteremia") AND (predict* OR "risk model" OR "predictive model" OR "prognostic model" OR "early prediction" OR "early detection") |

Supplementary Table 3 Forest plot of the random effects meta-analysis of pooled AUC estimates of models for mortality

| Stage | Model number | Pool AUC | 95% CI | *P* | I^2^ |
| --- | --- | --- | --- | --- | --- |
| Train | 12 | 0.800 | (0.761 - 0.838) | <0.001 | 97.9% |
| Validation | 9 | 0.778 | (0.751 - 0.804) | 0.005 | 63.5% |
| Test | 6 | 0.815 | (0.780 - 0.850) | 0.001 | 75.7% |
| AUC, area under the receiver operating characteristic curve; CI, confidence interval. | | | | | |

| 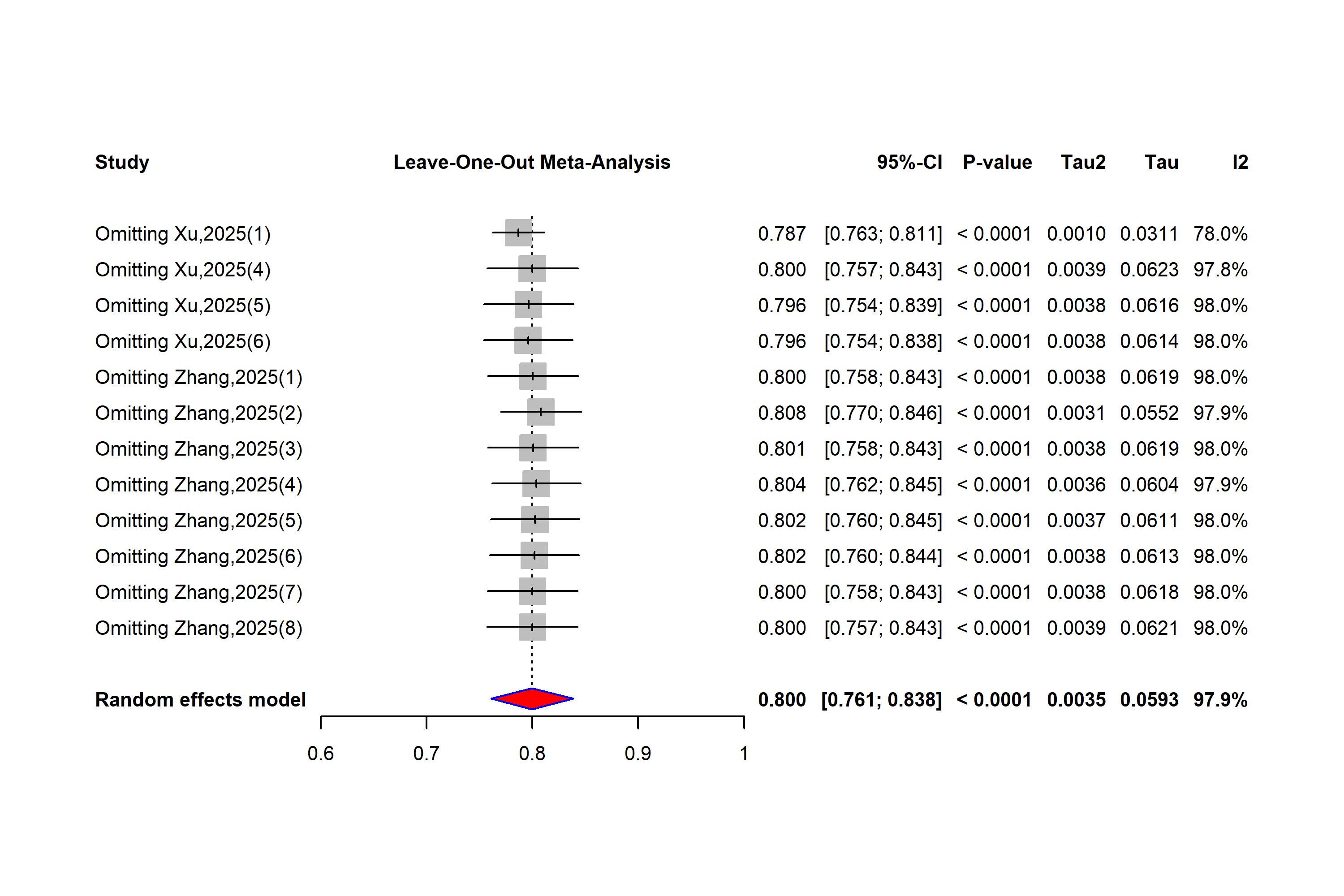 |
| --- |

Supplementary Figure 1 Sensitivity analysis of train models for mortality

| 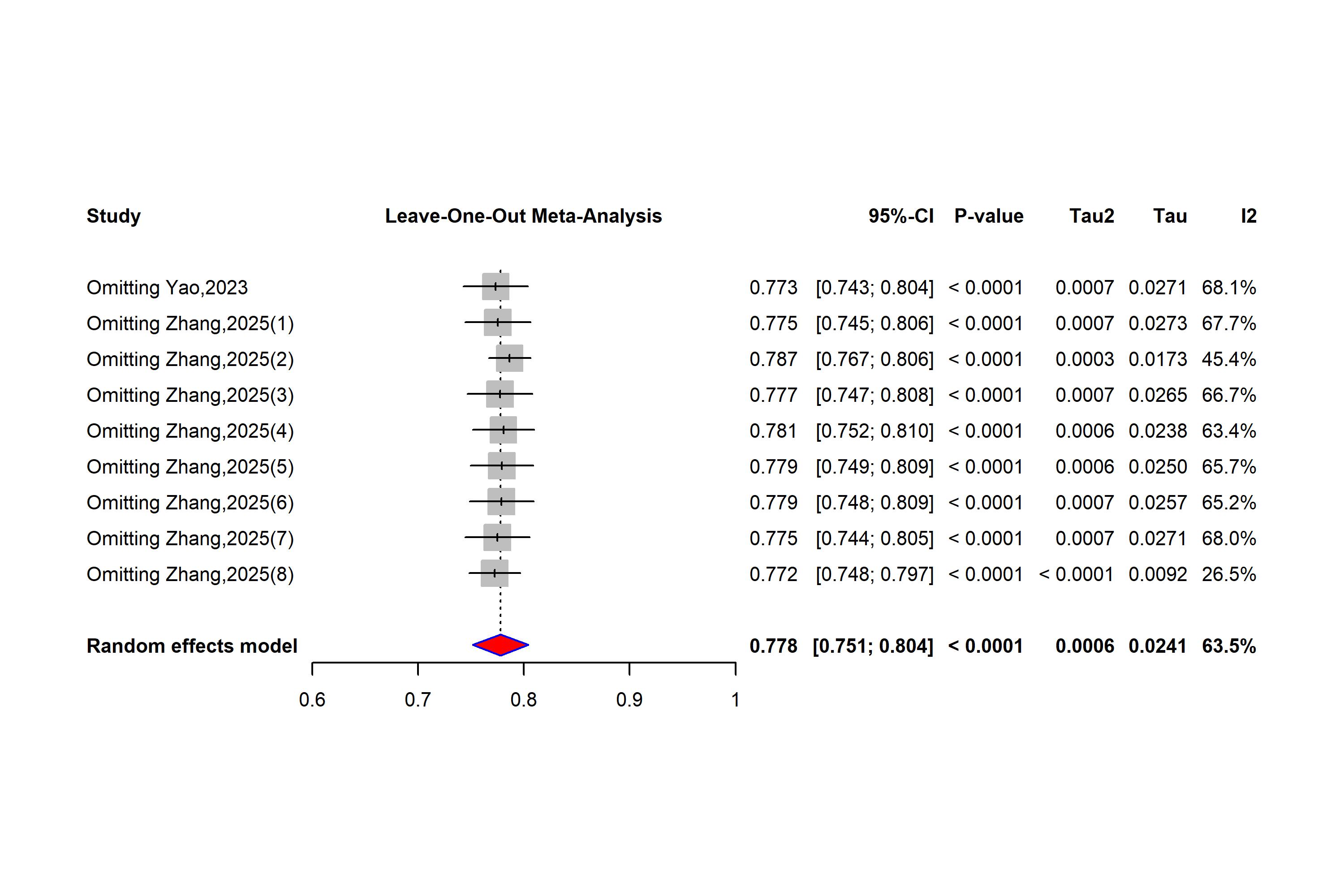 |
| --- |

Supplementary Figure 2 Sensitivity analysis of validation models for mortality

| 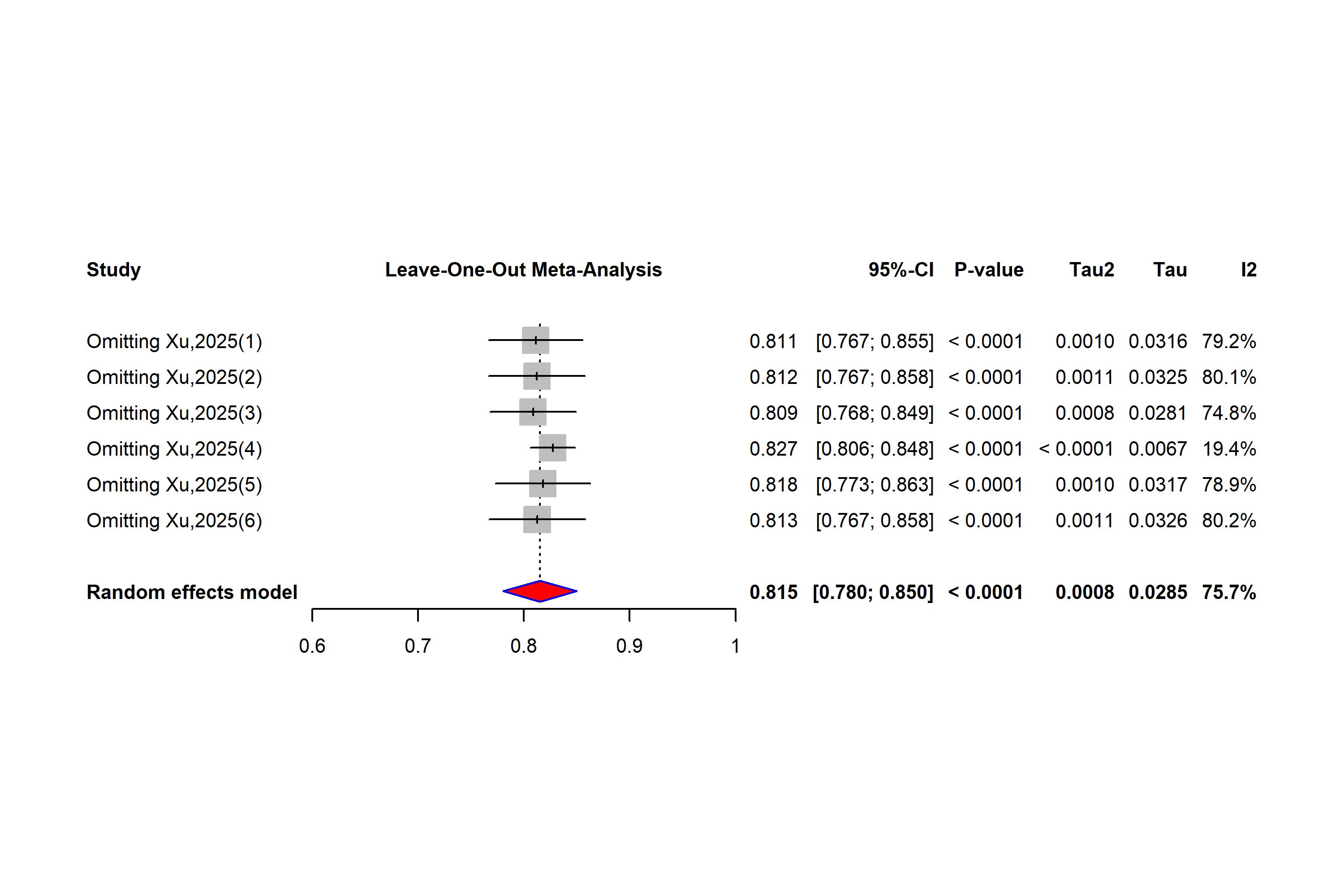 |
| --- |

Supplementary Figure 3 Sensitivity analysis of test model for mortality

| 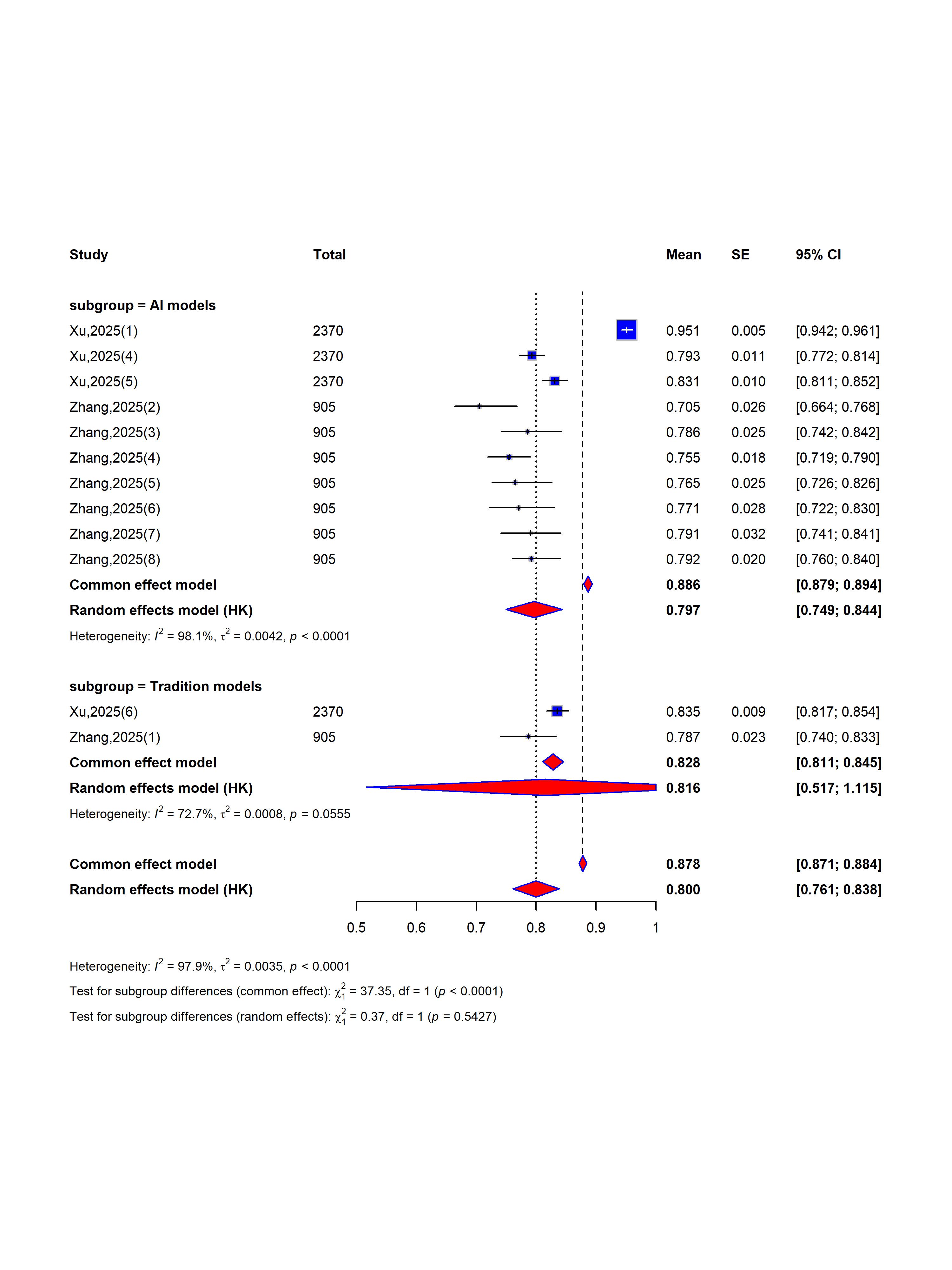 |
| --- |

Supplementary Figure 4 Subgroup analysis of train model for mortality

Supplementary Table 4 GRADE evidence summary for mortality prediction

| Type | | Train | Validation | Test |
| --- | --- | --- | --- | --- |
| No. of models | | 12 | 8 | 6 |
| No. of studies | | 2 | 1 | 1 |
| Quality assessment | ROB | Serious | Serious | Serious |
|  | Inconsistency | Serious | Serious | Serious |
|  | Indirectness | Serious | Serious | Serious |
|  | Imprecision | No | No | No |
|  | PB | No | No | No |
| No. of patients | | 3,275 | 100 | 1,016 |
| Results | Sensitivity (95% Cl) | 0.729 (0.682 - 0.777) | 0.681 (0.655 - 0.707) | 0.742 (0.696 - 0.789) |
|  | Specificity (95% Cl) | 0.807 (0.752 - 0.861) | 0.801 (0.759 - 0.842) | 0.777 (0.710 - 0.844) |
|  | AUC (95% Cl) | 0.800 (0.761 - 0.838) | 0.778 (0.751 - 0.804) | 0.815 (0.780 - 0.850) |
| Quality of the evidence | | Low | Low | Low |
| ROB, risk of bias; PB, publication bias; CI, confidence interval. | | | | |

Supplementary Note 1 Overview of core algorithms

This section outlines twelve foundational algorithms. While Logistic Regression (LR) serves as a baseline generalized linear model offering direct probabilistic interpretations, the remaining eleven fall into three broad categories: traditional distance- or probability-based models (KNN, NB, SVM); tree-based ensemble methods utilizing boosting and bagging (CART, RF, AdaBoost, GBDT, XGBoost, LightGBM); and neural network architectures (MLP, DNN).

1. AdaBoost (Adaptive Boosting)

AdaBoost relies on an iterative process of learning from previous errors. It builds a strong classifier by combining multiple weak learners, typically simple decision stumps. In each round, the algorithm increases the weights of previously misclassified samples, forcing the subsequent learner to focus on harder cases. The final prediction relies on a weighted majority vote based on each learner's accuracy ^[1]^.

1. DNN (Deep Neural Network)

DNNs extend traditional neural networks through multiple hidden layers, enabling the extraction of highly abstract features from raw data. Training relies on forward and backward propagation data passes through layers of linear transformations and non-linear activations (such as ReLU) to produce predictions, after which backpropagation updates the weights and biases using the chain rule to minimize loss ^[2]^.

1. DT / CART (Decision Tree / Classification and Regression Tree)

CART is a non-parametric algorithm that builds binary decision trees through recursive partitioning. It splits datasets by minimizing squared error for regression tasks or using the Gini Index for classification. To prevent overly complex structures that overfit training noise, CART applies post-pruning techniques, which penalize the number of leaf nodes ^[3]^.

1. GBT / GBDT (Gradient Boosting Tree)

GBDT modify the boosting approach by fitting new models to the residual errors of the current ensemble, rather than adjusting sample weights. In each iteration, a new regression tree is trained to predict the difference between the actual values and the model's current predictions. By incrementally reducing this gap, GBDT achieves high accuracy ^[4]^.

1. KNN (k-Nearest Neighbors)

As a classic "lazy learning" method, KNN does not involve an explicit training phase, instead storing the entire dataset. During inference, it assigns labels based on a majority vote from the K closest data points to a given query sample, typically measured by Euclidean distance. Its accuracy depends heavily on the chosen value of K and the specific distance metric used ^[5]^.

1. LightGBM (Light Gradient Boosting Machine)

LightGBM addresses the computational bottlenecks traditional GBDTs face with massive datasets. It accelerates training without losing predictive accuracy by implementing Gradient-based One-Side Sampling (GOSS) and Exclusive Feature Bundling (EFB). Additionally, it uses a leaf-wise rather than level-wise tree growth strategy, optimizing loss reduction by focusing on the most informative nodes ^[6]^.

1. LR (Logistic Regression)

Logistic Regression maps a linear combination of features to a probability between 0 and 1 using a logistic (sigmoid) function. Typically optimized via Maximum Likelihood Estimation (MLE), LR remains widely used in fields like clinical research due to its high interpretability and strong performance on linearly separable data ^[7]^.

1. MLP (Multilayer Perceptron)

The MLP is a foundational feedforward neural network comprising an input layer, one or more hidden layers, and an output layer. Non-linear activation functions (such as ReLU or Sigmoid) are applied at each layer to give the network its expressive power; without them, the architecture would merely perform a sequence of linear transformations ^[8]^.

1. NB (Naive Bayes)

NB applies Bayes' Theorem under the strong, often "naive," assumption that all features are conditionally independent. Although this assumption rarely holds perfectly in practice, the algorithm is highly effective and computationally efficient for high-dimensional problems like text classification. It estimates class probabilities by multiplying the conditional probabilities of individual features ^[9]^.

1. RF (Random Forest)

A prominent bagging method, Random Forest constructs an ensemble of independent CART trees. It achieves robustness and reduces model variance through a dual-randomization approach: bootstrap sampling of the training data (row sampling) and random feature selection at each split (column sampling). This diversity makes RF highly resistant to overfitting and noise ^[10]^.

1. SVM (Support Vector Machine)

SVM aim to find an optimal decision boundary, or hyperplane, that maximizes the margin between different classes. For datasets that are not linearly separable, SVMs employ the "kernel trick" to map inputs into a higher-dimensional feature space where a clear linear boundary can be established, without incurring prohibitive computational costs ^[11]^.

1. XGBoost (eXtreme Gradient Boosting)

XGBoost is a highly scalable and optimized implementation of gradient boosting. It directly integrates L1 and L2 regularization into the loss function to control model complexity and uses a second-order Taylor expansion to speed up convergence. With built-in mechanisms for handling sparse data and missing values, it is widely favored in both production environments and data science competitions ^[12]^.

Supplementary Reference

[1] Ding Y, Zhu H, Chen R, et al. An Efficient AdaBoost Algorithm with the Multiple Thresholds Classification [J]. Applied Sciences, 2022, 12(12): 5872.

[2] Barrios JP, Ansari MU, Olgin JE, et al. Multiview deep learning improves detection of major cardiac conditions from echocardiography [J]. Nature Cardiovascular Research, 2026, 5(3): 234-245.

[3] Ishwaran H. The effect of splitting on random forests [J]. Machine Learning, 2015, 99(1): 75-118.

[4] Friedman JH. Greedy function approximation: a gradient boosting machine [J]. The Annals of Statistics, 2001, 29(5): 1189-1232.

[5] Cover T, Hart P. Nearest neighbor pattern classification [J]. IEEE Transactions on Information Theory, 1967, 13(1): 21-27.

[6] Drin S, Kriuchkova A, Toloknova V. Predictive model for a product without history using LightGBM. Pricing model for a new product [J]. Scientific Notes of NaUKMA, 2023.

[7] Cristea DM, Sima I, Iantovics LB. Comparative analysis of optimized logistic regression with state-of-the-art models for complex gastroenterological image analysis [J]. Frontiers in Medicine, 2025, 12: 1655612.

[8] Rumelhart DE, Hinton GE, Williams RJ. Learning representations by back-propagating errors [J]. Nature, 1986, 323(6088): 533-536.

[9] Domingos P, Pazzani M. On the optimality of the simple Bayesian classifier under zero-one loss [J]. Machine Learning, 1997, 29(2/3): 103-130.

[10] Breiman L. Random forests [J]. Machine Learning, 2001, 45(1): 5-32.

[11] Cortes C, Vapnik V. Support-vector networks [J]. Machine Learning, 1995, 20(3): 273-297.

[12] Chen T, Guestrin C. XGBoost: a scalable tree boosting system [C]//Proceedings of the 22nd ACM SIGKDD International Conference on Knowledge Discovery and Data Mining. New York: ACM, 2016: 785-794.
